# Topographic patterns of retinal lesions in multiple evanescent white dot syndrome

**DOI:** 10.1007/s00417-023-06032-1

**Published:** 2023-03-29

**Authors:** Ariel Yuhan Ong, Johannes Birtel, Eleftherios Agorogiannis, Srilakshmi M. Sharma, Peter Charbel Issa

**Affiliations:** 1 Oxford Eye Hospital, Oxford University Hospitals NHS Foundation Trust, Oxford, UK; 2 Nuffield Laboratory of Ophthalmology, Nuffield Department of Clinical Neurosciences, University of Oxford, Oxford, UK; 3 Department of Ophthalmology, University Medical Center Hamburg-Eppendorf, Hamburg, Germany

**Keywords:** Multiple evanescent white dot syndrome, Fundus autofluorescence, Optical coherence tomography, Angiography, Multimodal imaging, Myopia

## Abstract

**Purpose:**

To demonstrate different topographic distributions of multiple-evanescent white dot syndrome (MEWDS) and secondary MEWDS disease and to describe possible associations.

**Methods:**

Clinical evaluation and multimodal retinal imaging in 27 subjects with MEWDS (29 discrete episodes of MEWDS). Ophthalmic assessment included best-corrected visual acuity testing and multimodal retinal imaging with OCT, blue-light autofluorescence, fluorescein and indocyanine green angiography, fundus photography, and widefield pseudocolor and autofluorescence fundus imaging.

**Results:**

The topographic distribution of MEWDS lesions was centered on or around the optic disc (*n* = 17, 59%), centered on the macula (*n* = 7, 24%), sectoral (*n* = 2, 7%), or was indeterminate (*n* = 3, 10%). The MEWDS episodes either occurred in the absence (‘primary MEWDS’; *n* = 14, 48%) or presence of concurrent chorioretinal pathology (‘secondary MEWDS’; *n* = 15, 52%). In patients with the latter, MEWDS lesions were often centered around a coexisting chorioretinal lesion. The majority of patients in both groups experienced resolution of their symptoms and retinal changes on multimodal imaging by 3 months.

**Conclusions:**

Distinct distributions of MEWDS lesions were identified. MEWDS may occur in tandem with other chorioretinal pathology, which may impact the topography of MEWDS lesions.

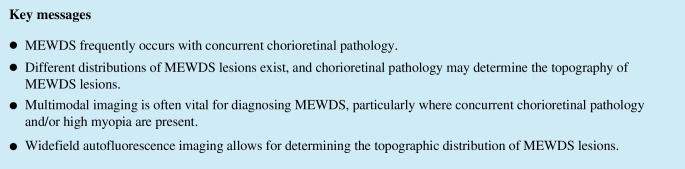

## Introduction

MEWDS was first described in 1984 as a unilateral, acute-onset, evanescent condition that presents with small clusters of discrete white dots most prominent in the perifoveal region and associated with a granular appearance of the fovea [1]. It was previously thought to be a primary choriocapillaritis [2], but more recent multimodal imaging studies suggest the retinal pigment epithelium (RPE)-photoreceptor complex to be the primary site of insult [3–5].

Classically, MEWDS is thought to occur in younger female patients, several of whom report a viral prodrome. However, an infectious or autoimmune etiology has yet to be elucidated and no associated systemic diseases have consistently been found [1, 6]. More recent reports have detailed a subset of cases with ‘MEWDS-like’ reactions, occurring contemporaneously with other ocular pathology which may result in exposure of retinal, RPE, and the Bruch membrane antigens to the immune system [7–13]. Various other descriptive terminology has been used for such ‘MEWDS-like’ reactions, including secondary MEWDS, epiMEWDS, and acute retinopathy [7, 8, 14]. While most affected patients experience complete visual recovery [6], others have protracted symptoms [15], which may be associated with the development of peripapillary atrophy, multifocal pigmentary changes, and/or acute zonal occult outer retinopathy (AZOOR) [16].

Here, we aim to further expand on these findings by illustrating different topographic distributions of MEWDS and/or MEWDS-like reactions that we have observed in our clinical practice in order to improve our understanding of its clinical manifestations.

## Methods

Subjects for this retrospective cohort study were identified at the Oxford Eye Hospital, UK, over the period January 2016 to January 2023. Participants were identified from an audit of the departmental electronic medical record system (Medisoft, Leeds, UK), which was cross-referenced with records kept by 2 retinal specialists (PCI and SMS). All clinical examinations were performed as part of routine clinical care, and therefore this work did not require formal ethical review [17]. The study was conducted in adherence with the Declaration of Helsinki.

Patients were included if they met diagnostic criteria for MEWDS, which included multifocal gray-white chorioretinal spots with foveal granularity, hyperfluorescent dots in the early phase of fluorescein angiography (FFA), hypofluorescent dots in the late phase on indocyanine green angiography (ICGA), and ellipsoid zone disruptions on spectral domain optical coherence tomography (OCT) [18].

Clinical records were reviewed for demographic information (age, gender, and ocular co-pathology) and clinical presentation (visual acuity, main presenting complaint, and duration of symptoms). Multimodal retinal imaging was obtained, which included OCT and fundus autofluorescence (AF) imaging (both HRA + OCT, Heidelberg Engineering, Heidelberg, Germany), fundus photography (Topcon, Tokyo, Japan), ultra-widefield pseudo-color and AF fundus imaging (Optos, Dunfermline, UK), FFA, and ICGA (both, Heidelberg Engineering and/or Optos) [19].

To determine the topography of retinal lesions, a horizontal and vertical line (maximum width) was drawn through the area of the chorioretinal spots on ultra-widefield AF imaging; the intersection of these lines defined the center of the total area of MEWDS lesions (at or around the optic disc, macular, or sectoral). If ultra-widefield AF imaging was not available, multifield late-phase ICGA and/or ultra-widefield pseudo-color images were used. Three authors (AYO, JB, and PCI) discussed each case and determined the topographical distribution via consensus.

For statistical analysis, continuous data were described with the median and interquartile range (IQR). As the Shapiro–Wilk test demonstrated a non-normal distribution, the Mann–Whitney *U* test was used to compare nonparametric data (age at presentation) at a significance level of *p* < 0.05.

## Results

### Description of patient cohort

We analyzed 29 MEWDS events in 27 patients (17 female and 10 male) with a median age of 36 years (interquartile range [IQR] 25 to 51). All patients had unilateral disease, and 11 patients (39%) were high myopes (≥ − 6 diopters). Patient characteristics are summarized in Table 1.

**Table 1:** Demographic and clinical features of the cohort of patients with multiple evanescent white dot syndrome (MEWDS)

ID	Age at Onset (years)	Gender	Eye	BCVA at Presentation (LogMAR)	Time to Presentation (Days)	Follow up (months)	BCVA at last follow-up (LogMAR)	Main Presenting Complaint			Ocular Comorbidities		Topographic Distribution of Lesions
								Scotoma	Photopsia	Blurred central vision	Myopia	Chorioretinal Pathology	
1	23	Male	Left	0.3	1	6.5	–0.2	Yes	–	–	–	–	Disc
2	23	Male	Left	0.2	3	21.2	–0.2	Yes	–	–	–	–	Disc
3	44	Female	Right	0.1	28	1.6	0.0	–	–	Yes	–	Punctate chorioretinal scarring in nasal and peripapillary retina	Macula
4	24	Male	Right	–0.2	2	3.8	0.0	–	Yes	–	–	–	Macula
5	29	Female	Right	0.1	4	1.5	0.1	Yes	–	–	–	–	Disc
6	36	Female	Right	0.2	3	38.4	0.1	Yes	Yes	–	–	–	Macula
7	48	Female	Left	–0.1	21	0.4	0.1	Yes	Yes	–	High myopia	–	Unclear
8	13	Female	Right	0.2	21	13.1	0.2	Yes	–	–	–	–	Disc
9	24	Female	Left	0.1	1	1.6	0.1	Yes	–	–	–	–	Sectoral
10	53	Male	Right	0.3	10	15.7	0.0	Yes	–	–	High myopia	Retinopexy to areas of lattice degeneration	Disc
11	61	Male	Left	0.0	7	7.5	0.0	Yes	–	–	–	Peripapillary atrophy, Macular chorioretinal scar	Disc
12	21	Male	Right	0.5	3	0.5	NR	Yes	–	–	–	–	Macula
13	56	Female	Left	**–**0.1	14	8.7	0.3	Yes	–	–	Moderate myopia	Patchy chorioretinal atrophy in peripapillary region	Disc
14	41	Female	Right	0.2	14	12.5	1.0*	–	Yes	–	High myopia	Myopic choroidal neovascular membrane, Lacquer cracks	Disc (first), Macula (second), Both (third)
15	71	Male	Left	0.2	7	10.5	0.1	–	–	Yes	–	Subfoveal RPE changes related to previous CSC	Disc
16	28	Female	Left	0.0	1	7.7	0.1	Yes	Yes	–	High myopia	–	Unclear
17	34	Male	Left	0.0	56	12.6	0.1	–	Yes	–	High myopia	Punctate inner choroiditis	Macula
18	54	Female	Left	0.0	5	3.2	0.1	Yes	–	–	High myopia	–	Unclear
19	62	Female	Left	1.3	2	1.5	1.0*	–	Yes	Yes	High myopia	Lacquer cracks	Disc
20	38	Male	Left	0.0	4	8.2	0.2	Yes	–	–	–	Angioid streaks	Disc
21	26	Female	Right	0.2	3	6.5	0.0	–	Yes	Yes	High myopia	Lacquer cracks	Disc
22	24	Female	Left	0.4	6	4.5	0.0	Yes	–	Yes	–		Disc
23	38	Female	Left	0.0	Unclear	3.1	0.0	Yes	Yes	–	High myopia	Punctate inner choroiditis	Sectoral
24	41	Male	Right	0.5	4	1.5	0.0	–	–	Yes	High myopia	Lacquer cracks	Macula
25	64	Female	Left	0.2	1	6.4	0.1	–	–	Yes	High myopia	Lacquer cracks	Disc
26	32	Female	Right	0.2	3	0.4	0.0	–	–	Yes	–	–	Disc
27	31	Female	Right	0.4	1	0.4	0.0	–	–	Yes	–	–	Disc

*Two eyes had a poor final LogMAR VA. This was related to structural changes from myopic choroidal neovascularization and atrophic changes from lacquer cracks, and not structural changes from MEWDS (which resolved satisfactorily). NR=not recorded. CSC=central serous chorioretinopathy

Thirteen patients (13/27, 48%) had concurrent chorioretinal pathology, including chorioretinal scarring or atrophy (*n* = 3), lacquer cracks with (*n* = 3) or without (*n* = 2) myopic choroidal neovascular membrane, punctate inner choroiditis (PIC) (*n* = 2), angioid streaks (*n* = 1), previous central serous chorioretinopathy (CSC) with residual RPE changes (*n* = 1), and previous laser retinopexy (*n* = 1). Patients with concurrent chorioretinal pathology tended to be older at presentation than those without (median 44 [IQR 38–62] vs 26 [23–33] years, *p* < 0.001).

Recurrence of MEWDS was documented in 1 patient (#14; 3 distinct episodes within 2 years), which was associated with contemporaneous expansion of myopic lacquer cracks. Another two patients recalled a previous diagnosis of MEWDS at different centers (one 3 years ago and another 21 years before); however, no confirmatory records were available, and their symptoms reportedly resolved spontaneously without treatment. A fourth patient experienced photopsia and a temporal scotoma 2 years prior to presentation, which resolved over a 2-week period. This had been attributed to a posterior vitreous detachment (PVD) at the time, and there was no multimodal imaging from this episode to clarify whether this could have been MEWDS-related.

### Topographical distribution of MEWDS lesions

**Fig. 1:**
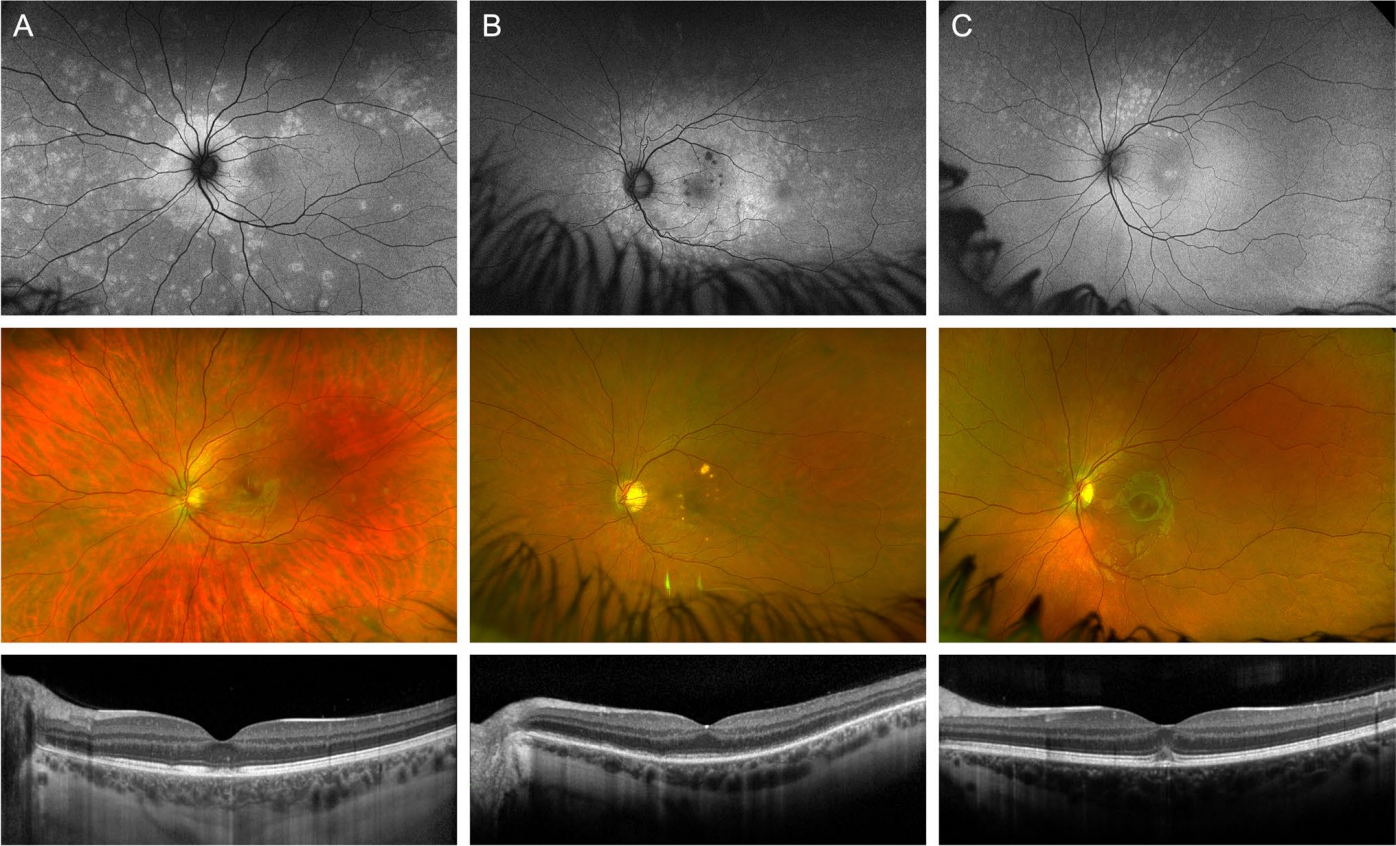
Widefield fundus autofluorescence images illustrate three distinct topographical distributions of MEWDS lesions: lesions centered on the optic disc (**A**), centered on the macula (**B**), and lesions with a sectoral distribution (**C**). Notably, the patient in B had a concurrent chorioretinal pathology—macular punctate inner choroidopathy scars

Multimodal imaging revealed different topographical distributions of MEWDS lesions, which were best illustrated on ultra-widefield AF imaging (Fig. 1). The most common distribution was characterized by MEWDS lesions that were densest or most confluent around the optic disc (17 of 29 events, 59%). The variable area involving more eccentric lesions seemed to be centered on or around the optic disc. In a subset of eyes in this group, the macula was relatively spared. In 7 out of 29 events (24%), MEWDS lesions were centered on the macula, and 2 eyes (7%) presented with a sectoral distribution. The pattern could not be conclusively ascertained in the remainder (3/29, 10%) because high-quality widefield images were not available. Involvement or sparing of the fovea may be observed in any subtype, including foveal involvement in cases with sectoral lesions that otherwise spare the central retina (Fig. 1C) or relative fovea sparing in patients with MEWDS lesions centered on the macula (Fig. 1B).

Patients without identifiable concurrent chorioretinal pathology (14/27, 52%) had MEWDS lesions with a range of topographical distributions: centered on or around the optic disc (7/14, 50%), centered on the macula (3/14, 21%), and sectoral (1/14, 7%). For the remainder (3/14, 21%), the distribution could not be determined due to the absence of widefield imaging.

**Fig. 2:**
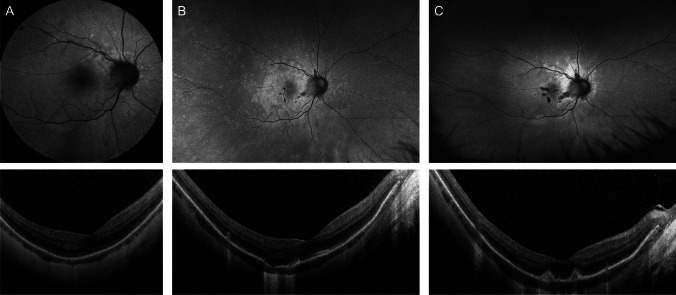
Patient (#14) with 3 documented episodes of MEWDS. The first episode showed dots centered on the disc and was associated with an expanding lacquer crack superior to the disc (**A**); the second was centered on the macula, with an expanding lacquer crack in the macula (**B**); the third was centered on the disc and was associated with an expanding lacquer crack inferotemporal to the disc (**C**).

In patients with concurrent chorioretinal pathology (13/27, 48%), the topographical distribution of MEWDS lesions was frequently centered on the lesion that presumably triggered the MEWDS episode. For example, they were centered on the optic disc or its immediate surroundings in patients with juxtapapillary chorioretinal lesions or breaks in Bruch’s membrane and centered on the macula in the patient with macular chorioretinal lesions (Fig. 1B). In the patient with documented recurrence of MEWDS (#14), the first and third episodes were centered on the optic disc and were associated with expanding juxtapapillary lacquer cracks; the second was centered on the macula and occurred with an expanding lacquer crack within the macula (Fig. 2; second episode previously illustrated in detail in Ref. 13).

However, topographic relationships between concurrent chorioretinal pathology and MEWDS lesions were not always well-defined. One patient (#11) with a macular chorioretinal scar had dots centered on the optic disc; however, an unusually marked distribution of peripapillary atrophy was observed at the same time. This was also the case for another patient (#15) with subfoveal RPE disturbances attributed to previous CSC, who had dots (MEWDS lesions) centered on the disc along with significant peripapillary atrophy and a possible juxtapapillary defect in Bruch’s membrane. Neither of these patients were myopic nor had glaucoma, and it was unclear whether the topographical distribution of MEWDS lesions was primarily associated with the peripapillary changes or macular pathology. In another patient (#3), PIC-related chorioretinal scars were present in the nasal and peripapillary regions, with MEWDS lesions centered on the macula.

### Value of multimodal imaging

**Fig. 3:**
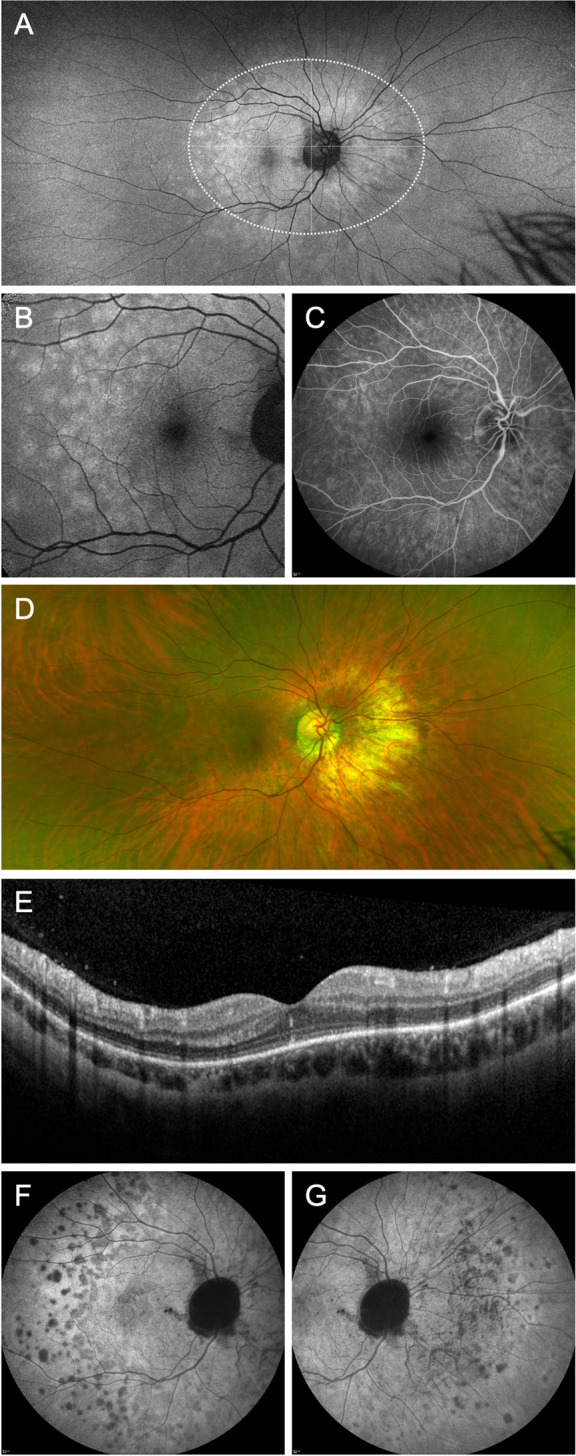
Multimodal imaging (patient #21) demonstrates the importance of different imaging modalities in diagnosing MEWDS, particularly in patients with high myopia. Widefield autofluorescence imaging (**A**) shows the lesions to be centered on the disc, which is not illustrated in 30° autofluorescence images (**B**). Fluorescein angiography (**C**) and widefield pseudo-color images (**D**) showed only faint lesions. Typical disruption of the ellipsoid band is shown on the macular OCT image (**E**). Late-phase indocyanine green angiography (30 − 40 min; **F**, **G**) shows hypocyanescent dots typical for MEWDS and highlights peripapillary breaks in Bruch’s membrane that are present concurrently. To determine the topography of retinal lesions, a horizontal and vertical line (maximum width) was drawn through the area of the chorioretinal spots on ultra-widefield AF imaging (A)

Widefield AF imaging was vital in visualizing the full extent of retinal lesions (Figs. 1, 2, and 3). Figure 3 illustrates that full topographic distribution was best captured on ultra-widefield AF imaging (A) but could not be ascertained on AF images limited to the posterior pole (B). Fluorescein angiography often did not add much information (C). The retinal changes in MEWDS were sometimes poorly visible on fundal examination (D), particularly in patients with myopic fundi. The combination of fundus AF and OCT (E) imaging was often invaluable in making the diagnosis. Moreover, potential underlying chorioretinal pathology was sometimes only visible on specific imaging modalities, such as the visualization of small breaks in Bruch’s membrane on late-phase ICGA images (F, G). In this example, late-phase ICGA images were also helpful for determining the nasal eccentricity of lesions and that hence, MEWDS lesions were approximately centered on the disc.

Patient #14, who had 3 distinct episodes of MEWDS, is another example illustrating the value of multimodal imaging when OCT images are difficult to interpret due to additional myopia-related pathology or mild disease (Fig. 2). The first episode featured only mild changes, which were not evident on pseudocolor images due to the appearance of her myopic fundus. The mild hyperautofluorescent dots and subtle photoreceptor layer disturbances were identified when reviewing her previous AF and OCT imaging after she presented with her second episode, due to the heightened index of suspicion.

### Clinical course

Patients were followed up for a median of 8 months (IQR: 2–16 months). Follow-up data of at least 3 months’ duration were available from 18/27 (67%) patients. Of these, the majority (14/18, 78%) experienced resolution of their symptoms and retinal changes on multimodal imaging by 3 months. Four patients had symptoms persisting beyond 12 months: two had dots centered on the disc and two on the macula at their first presentation. At their final visit, there were a few persistent scattered white dots on AF, alongside persistent mild peripapillary ellipsoid zone loss and chorioretinal atrophy on OCT imaging. Of these 4 patients, 1 developed an acute zonal occult outer retinopathy (AZOOR)-like picture with thinning of the outer nuclear layer in the peripapillary region at 4 months.

## Discussion

This study describes different topographical distributions of the fundus changes observed in MEWDS: centered on the macula, centered on the optic disc, or sectoral. Lack of multimodal retinal imaging, including widefield fundus autofluorescence and (very) late-phase ICG imaging, may explain why MEWDS fundus changes have previously been described as primarily affecting the perifoveal region. Widefield imaging is vital in visualizing the extent of retinal lesions, which may be poorly visible on fundal examination, particularly in myopic fundi [13].

Our series indicates that there may be an association between the topographical distribution of MEWDS lesions and coexisting chorioretinal pathology in some patients. The clinical significance of the topographical distributions in MEWDS and whether they carry any prognostic significance in respect of recurrence, recovery, development of AZOOR, or accelerated atrophy, remains to be elucidated. Based on our data, we cannot comment if different distribution patterns of MEWDS lesions may occur sequentially, as this would require prospective standardized recordings of high-quality wide-field autofluorescence images with short intervals. Of note, different patterns of distribution have also been described in other related conditions; for example, different patterns of spread in patients with AZOOR [20].

Almost half of our cohort had concurrent ocular co-pathologies involving the choroid and retina. This frequent co-occurrence mirrors recent studies, which described antecedent or concurrent ocular insults including choroidal neovascularization [10], angioid streaks [8], ocular trauma [9], and inflammatory conditions such as chorioretinitis [7]. A recent study that addressed the outcome of visual acuity in patients with MEWDS reported a similar coexistence of chorioretinal disease (31%; 21/68 patients) but excluded these patients from further analysis [6]. In our patient cohort, although the group with concurrent chorioretinal pathology was significantly older, both groups had similar retinal phenotypes without obvious difference in prognosis. Thus, we postulate that primary and secondary MEWDS are part of the same disease spectrum rather than a distinct entity.

It has been hypothesized that MEWDS develops from the complex interplay between genetic susceptibility and environmental triggers [21]. Environmental factors may include exposure to retinal antigens resulting from damage to the RPE-Bruch’s membrane interface, which may expose retinal antigens and trigger a local inflammatory response [7, 8], or potential associations with viral infections such as acute Epstein-Barr virus or herpes virus infections [22, 23]. However, the inherent problems with serum retinal autoantibody testing make it challenging to prove this theory [24, 25]. If such analysis becomes possible in the future, it might reveal specific ocular antigen(s), as seen in cancer- or melanoma-associated retinopathy, and may also help further our understanding of the similarities or differences in the pathogenesis of MEWDS and ‘secondary’ or ‘epi’-MEWDS.

We speculate that MEWDS may potentially be underdiagnosed, particularly in patients with atypical presentation and/or because comprehensive multimodal retinal imaging may not be performed if MEWDS is not suspected [26, 27]. Diagnostic difficulty may be further enhanced in patients with high myopia or due to confirmation bias in presence of concurrent chorioretinal pathology. For instance, photopsias in high myopes may also be attributed to nascent posterior vitreous detachment, and focusing on the concurrent chorioretinal pathology (such as a myopic CNV) may also distract from the diagnosis. As such, a higher index of suspicion in these patients may be helpful.

In conclusion, a heightened awareness of this retinal entity, its signs and symptoms, and a more rigorous use of multimodal imaging may help to improve the diagnosis of MEWDS as well as enhance our understanding of secondary MEWDS.

### Strengths and limitations

Limitations include the retrospective nature of the study and all this entails, including the lack of standardized review intervals and imaging protocols. It is also not possible to comment on whether topographical distribution may change from one to another and if patterns may overlap. Despite the small number of patients included, which is due to the relative rarity of the condition, this study ranks among the larger cohorts of MEWDS cases reported. Multicenter studies with standardized high-quality imaging protocols and serial imaging in the acute phases may add to our findings and further enhance our understanding of this condition and its pathogenesis. This may also allow more robust associations between phenotypic expression and other parameters, such as outcomes or predictors of recurrence to be drawn.
